# *Trans*-Stilbenes in Commercial Grape Juices: Quantification Using HPLC Approaches

**DOI:** 10.3390/ijms17101769

**Published:** 2016-10-24

**Authors:** Julia López-Hernández, Ana Rodríguez-Bernaldo de Quirós

**Affiliations:** Faculty of Pharmacy, Department of Analytical Chemistry, Nutrition and Food Science, University of Santiago de Compostela, Campus Vida, 15782 Santiago de Compostela, Spain; ana.rodriguez.bernaldo@usc.es

**Keywords:** *trans*-stilbenes, commercial fruit juices, high-performance liquid chromatography, variable wavelength detector, fluorescence detector

## Abstract

*Trans*-stilbenes belong to the group of polyphenolic phytoalexins, and occur in many plant foods. These compounds have received great attention by researchers due to their well-known beneficial health effects. In the present study a chromatographic method that comprises the use of variable wavelength (VWD) and fluorescence (FLD) detectors in series for the analysis of *trans*-stilbenes is presented. The relation of peak-area obtained with both detectors is proposed as an alternative and complementary approach for the rapid identification of these phenolic compounds. The proposed method was applied to determine *trans*-stilbenes in commercial fruit juices. *Trans*-piceid was the most common *trans*-stilbene found in the samples analyzed. The method was validated in terms of linearity, sensitivity and repeatability. Appropriate sensitivity and good linearity (*r*^2^ > 0.9991) were achieved.

## 1. Introduction

The *trans*-stilbenes belong to the group of the polyphenolic phytoalexins that are synthesized in plants as a response to stress; these compounds are naturally occurring in many fruits such as grapes [1,2,3] and berries [3,4,5], which are used in the production of commercial juices.

The interest and studies on these substances have increased due to the potential beneficial actions of the stilbenes in the prevention of oxidative stress and age-related diseases such as obesity, type 2 diabetes, cardiovascular and neurodegenerative diseases [6], and cancer risk [7].

Owing to the healthy effects attributed to these compounds, recent research has focused on the addition of these phenolic compounds in the food matrix; being considered excellent candidates as functional ingredient [8].

Different stilbenes have been identified in natural products including resveratrol, piceatannol, and pterostilbene, among others [9]. Piceatannol (also named astringin) is a stilbene that is present in different fruits including grapes, vaccinium berries and so on. As it has been commented on above. various positive effects on the human health have been recognized in this class of phenolic compounds, additionally they seem to be an excellent antioxidant, stronger even than resveratrol [10].

Since the level of these stilbenes in the plant samples is very low, sensitive techniques are required for their determination. Several procedures to extract these natural compounds from the matrix have been reported in the literature. Direct injection without previous treatment simplified and shortened analysis time [11]; solid-phase extraction is a good alternative when purification and concentration steps are required, the common C-18 cartridges have been extensively used with satisfactory results. A cartridge of the polymeric adsorbent polystyrene-divinylbenzene was employed for the analysis of all of the monomers of resveratrol and good results were obtained [12]. Although to a lesser extent liquid–liquid extraction have also been employed in the analysis of polyphenols [13].

Regarding the separation techniques, both liquid and gas chromatography (GC) have been applied. Numerous High Performance Liquid Chromatography (HPLC) methods with different detection systems [14,15,16,17,18,19,20] have been described.

Kolouchová-Hanzlíková et al. (2004) [14] propose the use of ultraviolet (UV) detection with electrochemical detection to analyze free resveratrol isomers in wine and plant extracts. Another approach that involves the use of chemiluminescent detection for the direct analysis of *trans*-resveratrol in red wine is described by Zhou et al. [16].

The UV and fluorescence detector in series provided high selectivity for the determination of different phenolic compounds including *trans*-piceid, *cis*-piceid, *trans*-resveratrol, *cis*-resveratrol as reported by Rodríguez-Bernaldo de Quirós et al. (2007) [15]. More recently, liquid chromatography coupled to mass spectrometry including triple quadrupole and quadrupole time-of-flight (QTOF) have been successfully applied [18,19,20].

Reversed-phase mainly C-18 columns and a mobile phase composed by an organic solvent, such as acetonitrile or methanol, water and an acidic modifier are the usual chromatographic conditions for the analysis of these compounds. UV and diode array detector (DAD) are frequently used; however, when high sensitivity and selectivity is essential, mass spectrometry is the detection system of choice.

Gas chromatography–mass spectrometry (GC-MS) has also been used but in general this technique involves a derivatization step previous to the GC analysis [13].

In the present study, a methodology that comprises the use of VWD and FLD detectors in series for the analysis of *trans*-stilbenes is presented. The relation of peak-area obtained with both detectors is proposed as an alternative and complementary method for the rapid identification of these phenolic compounds. Furthermore, commercial grape juices were analyzed directly without previous treatment.

## 2. Results and Discussion

### 2.1. Chromatographic Method and Performance Characteristics

*Trans*-stilbenes are natural compounds occurring in different foods, plants, etc. In order to characterize the phenolic profile of these natural products, simple, rapid and reliable methods are required. In this study, a simple approach to identify *trans*-stilbenes in commercial fruit juices was proposed. First of all, preliminary assays were performed with the aim to optimize the chromatographic conditions. Several column temperatures (25, 30, and 35 °C) and flow rates (0.4, 0.5 and 0.6 mL/min) were tested. An appropriate peak resolution was obtained with the column thermostated at 30 °C and using a flow-rate of 0.5 mL/min. Secondly, and once the analytical conditions were fixed, the performance characteristics of the proposed method were studied.

The linearity of the method was tested using a series of *trans*-resveratrol, *trans*-piceatannol *trans*-pterostilbene and *trans*-piceid standard solutions of known concentration. The calibration curves were constructed using seven concentration levels and they were fitted to a linear equation. Each point of the calibration curve is the average of three peak-area measurements. Parameters of linearity, range of linearity, origin ordinate, slope and determination coefficients, are presented in Table 1. The proposed method showed good linearity within the range of concentrations studied. The coefficients of determination obtained were in all cases ≥0.9991.

The detection and quantification limits (LOD and LOQ), defined as a signal three and ten times higher than noise level, respectively) calculated in accordance with the Analytical Chemical Subcommittee guidelines [21] are shown in Table 1. The method exhibited a good sensitivity, the results obtained were slightly lower than those reported by other authors [22]. The VW detector showed better sensitivity with LOD and LOQ lower than those obtained with FLD.

The precision was determined by analyzing six replicates of the standards at a concentration of 0.2 µg/mL, and expressed as the percentage of RSD (RSD% (*n* = 6)) (Table 1). Repeatabilities lower than 3.3% were obtained for all analytes. Best results were obtained with VWD compared with FLD.

As can be inferred from Table 1, the relationship between the peak-areas obtained with both detectors VWD/FLD in the seven concentration levels of standard solutions analyzed present constant values with a coefficient of variation (*n* = 21) (CV%) lower than 4% for the four *trans*-stilbenes studied, so this relationship could be used as a criterion for identification purposes.

### 2.2. Determination of Stilbenes in Commercial Grape Juices

**Figure 1:**
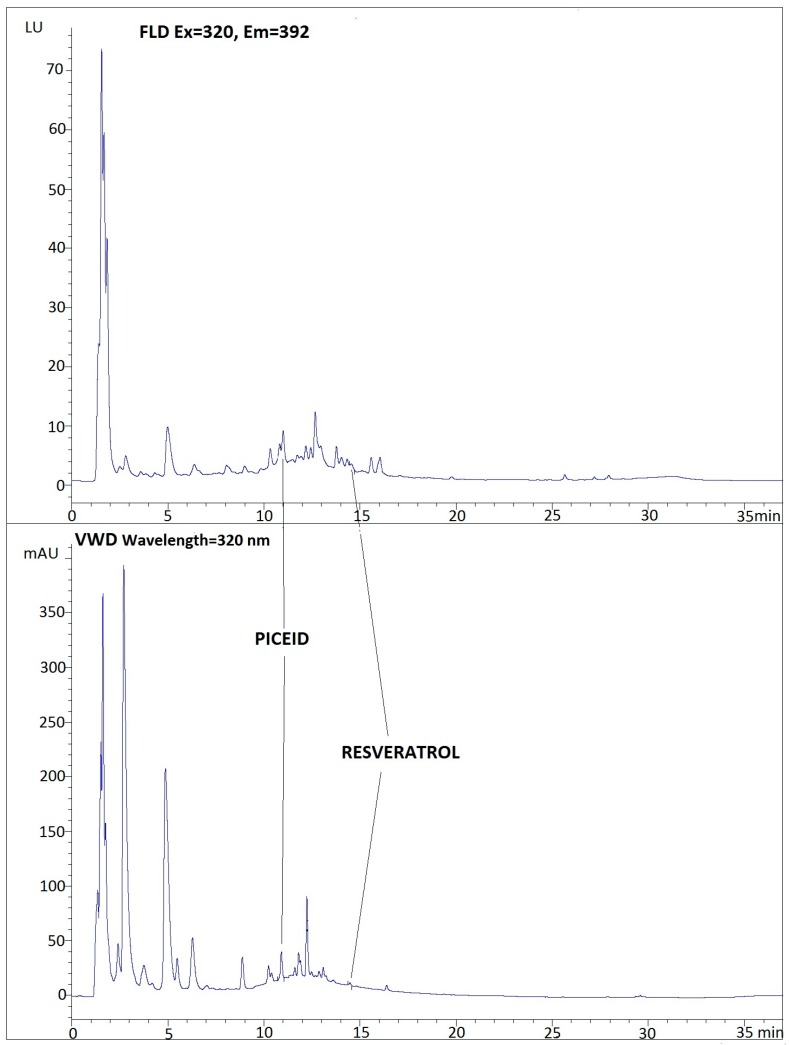
Chromatogram of a commercial fruit juice (Sample 5).

The results of the determination of *trans*-stilbenes by HPLC, with the two different types of detectors, are shown in Table 2. A chromatogram of sample is presented in Figure 1.

The most common *trans*-stilbene in the juices analyzed was *trans*-piceid, which was found in ten of eleven samples, the concentrations determined ranged between 0.29 ± 0.02 and 6.59 ± 0.45 μg/mL. *Trans*-piceatannol was only found in Sample 2 (0.26 ± 0.01 μg/mL), which corresponds, according to the packaging information, to grape juice made from concentrate 99%. Nevertheless, higher amounts were detected by Boue et al. [23] in sugarcane juice (8.5 ± 1.7 mg/L). Rodríguez-Cabo et al. [9] detected *trans*-piceatannol at a concentration of 496 ± 12 ng/mL in red wine Mencia variety. Five juices samples contained *trans*-resveratrol at a concentration level varying from 0.15 ± 0.01 to 0.79 ± 0.05 μg/mL. In general, these concentrations were lower than those found in European red wines. Buiarelli et al. [22] and Stecher et al. [24] reported levels found in Italian wines and chianti, respectively. In addition, high amounts of the *trans*-stilbene were found in Spanish wines, particularly in Mencia variety [20]; in Portuguese and Azores Island wines [25]; and in wines from Greek [26]. *Trans*-pterostilbene was not detected in any of the samples analyzed.

High-performance liquid chromatography-photodiode array detector-mass spectrometry detector (HPLC-PAD-MS/MS) was used to confirm the presence of *trans*-piceid, *trans*-piceatanol and *trans*-resveratrol in grape juices.

The deprotonated molecular ions [M − H]^−^ of the *trans*-stilbenes were selected as the precursor-ions. The identifications of the main products obtained are shown in Table 3. Some of the main products have been previously reported by Buiarelli et al. [23].

The regression analysis performed on the obtained data of *trans*-stilbenes by HPLC with fluorescence and variable wavelength detector show that the *p*-value in the ANOVA table is less than 0.01, indicating that there is a statistically significant relationship between both methods for a confidence level of 99%. According to the statistic *r*^2^, this model explains the 99.80% of the variability in HPLC and has a correlation coefficient equal to 0.9990, which indicates a strong correlation between variables.

## 3. Materials and Methods

### 3.1. Samples

Different commercial packs of grape juices mainly containing grape juice (1–6), grape juice 65% with pomegranate and blackcurrant juice (7–9), grape and pomegranate juice 50% (10) and grapes juice 92% with strawberry and cranberry juice (11) were purchased in local supermarkets of Santiago de Compostela in June 2016. They were immediately analyzed and following kept cold in a refrigerator for further assays. The composition indicated in the labeling of the different packaging of juice samples is presented in Table 4.

### 3.2. Reagents and Standard Solutions

Methanol and acetonitrile gradient grade for liquid chromatography were supplied by Merck (Darmstadt, Germany). Water used for all solutions was obtained from a Milli-Q water purification system (Millipore) (Woburn, MA, USA). Acetic solution 50% for HPLC and standards of *trans*-resveratrol, *trans*-piceatannol *trans*-pterostilbene and *trans*-piceid were purchased from Sigma-Aldrich (St. Louis, MO, USA). All standards presented a purity ≥95%. The chemical structures of the molecules are presented in Table 5. The stock solutions were prepared in methanol and standard solutions by diluting with water.

### 3.3. Identification and Quantification

Chromatographic peaks were identified by comparing their retention times, spectra data of fluorescence (Ex spectra 200–360 nm, Em 392 nm) and by means of the relationship between areas VW/FL with those of the pure standards, and by spiking the samples with standard solutions. Quantification was performed on the basis of linear calibration plots of peak area (*n* = 3) against concentration. Calibration lines were constructed based on seven concentration levels of standard solutions.

### 3.4. Preparation of Samples

For HPLC analysis, samples of drinks were filtered (hydrophilic membrane filter 0.45 µm PTFE (Membrane solutions LLC, Shanghai, China) and analyzed directly, in triplicate. Throughout the entire process, samples were protected from UV light.

### 3.5. Instrumentation and Method Conditions

#### 3.5.1. High Performance Liquid Chromatography (HPLC) Method

The standards and samples were analyzed using an HP1100 chromatography system (Hewlett-Packard, Waldbronn, Baden-Württemberg, Germany) consisted of a quaternary pump, degassing device, autosampler, variable wavelength detector, fluorescence detector and an Agilent ChemStation for LC and LC/MS systems software. The injection volume was 20 μL. The separation of the compounds was performed on a Kinetex EVO C18 100A column (150 mm × 3 mm, 5 µm) (Phenomenex) at 30 °C with a column thermostating system (Spectra Physics 8792, San Jose, CA, USA). The chromatographic conditions of the method were selected according to Rodríguez-Bernaldo de Quirós et al. [27] with modifications. The mobile phase consisting of water/acetic acid (99:1 *v*/*v*) as solvent A, water/acetonitrile/acetic acid (67:32:1 *v*/*v*/*v*) as solvent B and acetonitrile as solvent C, at a flow of 0.5 mL/min. The gradient program was as follows: 0 min, 80% A, 20% B; 18 min, 100% B; 28 min, 100% C; 33 min, 100% B and 37 min, 80% A, 20% B. The compounds were detected at 320 nm when using UV detection and the FLD detector was set at λem 392 nm and λex 320 nm.

#### 3.5.2. HPLC-PDA-MS/MS as a Confirmatory Technique

To confirm the identification of stilbenes in food samples an HPLC-PDA-MS/MS system consisted of an Accela Autosampler, an Accela 1250 pump with degasser and two detectors in series: PDA (Photodiode Array Detector) coupled to a TSQ Quantum Access max triple-quadrupole mass spectrometer controlled by Xcalibur software (Thermo Fisher Scientific, San Jose, CA, USA) was used. The mass spectrometer operated in negative Electrospray Ionization mode (ESI). The chromatographic conditions, were the same as in the HPLC analysis. *Trans*-stilbenes were characterized by its retention time relative to an external standard, PDA spectrum (200–600 nm), precursor ion and fragmentation ions obtained. The gas used was nitrogen, at a pressure of 35 psi. The spray voltage was 2500 V and the vaporization and capillary temperatures were set at 400 °C and 380 °C, respectively. The detection was carried out in a Single Reaction Monitoring (SRM) and a mass range between 140 and 400 *m*/*z*.

#### 3.5.3. Statistical Analysis

Statgraphics-Plus 5.1 (Statpoint Technologies Inc., Warrenton, VA, USA) software was used to perform lineal simple regression analysis and ANOVA of the results obtained by HPLC with variable wavelength and fluorescence detector, to establish relationship between them. The level of significance was *p* < 0.01.

## 4. Conclusions

Briefly, in the present work, an approach based on the relationship of peak-area obtained by VWD and FLD is proposed to identify *trans*-stilbenes in complex matrix. The proposed method was applied to analyze these phenolic compounds in commercial fruit juices.

## Figures and Tables

**Table 1:** Retention times, relationship between areas, linearity parameters, detection and quantification limits (LOD, LOQ) and repeatability of the High Performance Liquid Chromatography (HPLC) system for standards.

Compound	Retention Time (min)	Area^VW^ Area^FL^	Intercept	Slope	*r*^2^	Range (µg/mL)	LOD (µg/mL)	LOQ (µg/mL)	RSD%
*Trans*-piceid (HPLC-FL)	10.9	3.35 ± 0.13	0.8219 ± 0.1191	50.30 ± 0.45	0.9993	0.12–4.8	0.0226	0.0752	3.2
*Trans*-piceid (HPLC-VW)	10.8		3.2061 ± 0.7037	169.90 ± 0.068	0.9994	0.12–4.8	0.007	0.0238	0.42
*Trans*-piceatannol (HPLC-FL)	12.1	1.25 ± 0.055	−22.768 ± 4.8633	214.84 ± 1.08	0.9995	0.1–4.0	0.009	0.0300	2.9
*Trans*-piceatannol (HPLC-VW)	12.0		−30.090 ± 1.2111	270.99 ± 0.88	0.9993	0.1–4.0	0.007	0.0223	2.1
*Trans*-resveratrol (HPLC-FL)	14.6	3.24 ± 0.061	−6.0551 ± 1.4745	127.00 ± 2.30	0.9995	0.02–5.0	0.010	0.0330	0.75
*Trans*-resv (HPLC-VW)	14.5		−14.880 ± 0.6842	406.85 ± 0.63	0.9996	0.02–5.0	0.003	0.0101	0.15
*Trans*-pterostilbene (HPLC-FL)	24.8	3.21 ± 0.14	−10.463 ± 0.0388	100.59 ± 0.24	0.9991	0.08–8.0	0.012	0.0406	2.4
*Trans*-pterostilbene (HPLC-VW)	24.7		−25.0530 ± 2.6570	328.96 ± 2.25	0.9995	0.08–8.0	0.005	0.0161	0.8

**Table 2:** Concentrations of *trans*-stilbenes (µg/mL) with fluorescence detector (FLD) and variable wavelength (VW) detectors in commercial grape juices, *n* = 3 replicates.

Samples	*Trans*-Piceid		*Trans*-Piceatannol		*Trans*-Resveratrol		*Trans*-Pterostilbene	
	FL	VW	FL	VW	FL	VW	FL	VW
1	0.29 ± 0.01	0.29 ± 0.02	n.d.	n.d.	n.d.	n.d.	n.d.	n.d.
2	6.56 ± 0.22	6.59 ± 0.45	0.25 ± 0.01	0.26 ± 0.01	0.27 ± 0.01	0.27 ± 0.02	n.d.	n.d.
3	1.24 ± 0.10	1.24 ± 0.10	n.d.	n.d.	0.83 ± 0.07	0.79 ± 0.05	n.d.	n.d.
4	1.42 ± 0.06	1.35 ± 0.06	n.d.	n.d.	0.16 ± 0.01	0.15 ± 0.01	n.d.	n.d.
5	1.31 ± 0.11	1.27 ± 0.10	n.d.	n.d.	0.16 ± 0.08	0.15 ± 0.01	n.d.	n.d.
6	0.85 ± 0.06	0.81 ± 0.05	n.d.	n.d.	n.d.	n.d.	n.d.	n.d.
7	1.25 ± 0.04	1.24 ± 0.04	n.d.	n.d.	n.d.	n.d.	n.d.	n.d.
8	1.47 ± 0.09	1.42 ± 0.06	n.d.	n.d.	n.d.	n.d.	n.d.	n.d.
9	0.52 ± 0.02	0.51 ± 0.02	n.d.	n.d.	n.d.	n.d.	n.d.	n.d.
10	n.d.	n.d.	n.d.	n.d.	0.36 ± 0.02	0.36 ± 0.02.	n.d.	n.d.
11	0.46 ± 0.02	0.41 ± 0.025	n.d.	n.d.	n.d.	n.d.	n.d.	n.d.

n.d. = not detected.

**Table 3:** Retention time, λ max, and mass spectrometry conditions of *trans*-stilbenes.

Molecule	PDA λ max.	Precursor Ion	Product Ions	Collision Energy	Tube Lens
*Trans*-piceid	308, 319	389	227	22	−67.08
			185	40	
			143	51	
*Trans*-piceatannol	306, 322	243	225	21	−68.83
			201	22	
			159	28	
*Trans*-resveratrol	306, 317	227	185	21	−65.83
			183	20	
			143	29	
*Trans*-pterostilbene	307, 319	255	240	22	−59.32
			197	32	
			169	35	

**Table 4:** Composition of grape juices.

Samples	Composition
1	Red grapes juice 100%
2	Grape juice made from concentrate 99%
3	Grape juice from concentrate
4	Red grape Merlot juice 100%
5	Red grape Merlot juice 100%
6	Organic grape juice made from concentrate
7	Red grape juice 65%, pomegranate juice 25%, blackcurrant juice 10%
8	Red grape juice 65%, pomegranate juice 25%, blackcurrant juice 10%
9	Red grape juice 65%, pomegranate juice 25%, blackcurrant juice 10%
10	Grape juice 50%, pomegranate juice 50%
11	Red grapes squeezed juice 92%, strawberry pure 4% and cranberry juice squeezed 4%

**Table 5:** Chemical structures name of the analytes studied.

Chemical Structure	Name	CAS	MW	Log P (octanol-water)
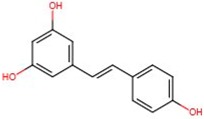	*Trans*-resveratrol	501-36-0	228.246	3.080
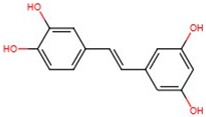	*Trans*-piceatannol	10083-24-6	244.2448	n.a.
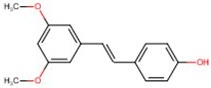	*Trans*-pterostilbene	537-42-8	256.299	n.a.
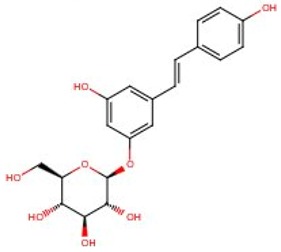	*Trans*-piceid	65914-17-2	390.386	n.a.

n.a.: not available, CAS: Chemical Abstracts Service, MW: Molecular weight.
